# Pharmacological Effects of Rutaecarpine as a Cardiovascular Protective Agent

**DOI:** 10.3390/molecules15031873

**Published:** 2010-03-15

**Authors:** Sujie Jia, Changping Hu

**Affiliations:** 1 Department of Pharmaceutics, The Third XiangYa Hospital of Central South University, Changsha 410013, China; E-Mail: sujiejia@yahoo.com; 2 Department of Pharmacology, School of Pharmaceutical Sciences, Central South University, Changsha 410078, China

**Keywords:** rutaecarpine, cardiac protection, vasodilator, anti platelet aggregation, anti inflammation

## Abstract

Many studies indicate that traditional Chinese herbs are beneficial in the prevention and treatment of cardiovascular diseases. *Evodia rutaecarpa* (‘Wu-Chu-Yu’) remains the most popular and multi-purpose herb traditionally used in China for treatment of headache, abdominal pain, postpartum hemorrhage, dysentery and amenorrhea. Rutaecarpine is one of the intriguing indolopyridoquinazoline alkaloids isolated from ‘Wu-Chu-Yu’. Rutaecarpine has been shown to have cardiovascular biological effects such as inotropic and chronotropic, vasorelaxant, anti-platelet aggregation and anti-inflammatory effects. Furthermore, it has been reported that rutaecarpine has beneficial effects on some cardiovascular diseases. This review summarizes data on the cardiovascular pharmacological actions of rutaecarpine the published over the recent years, aiming to provide more evidence supporting its use in the treatment of cardiovascular diseases.

## 1. Introduction

Rutaceous plants, especially *Evodia rutaecarpa* (whose dried fruit is named ‘Wu-Chu-Yu’ in China), have been widely used for the treatment of gastrointestinal disorders, headache, amenorrhea, and postpartum hemorrhage in traditional oriental medicine for hundreds of years [1,2]. It is also reported to have a remarkable central stimulant effect [3]. Rutaecarpine (8,13-dihydroindolo-[2',3':3,4]-pyrido [2,1-b] quinazolin -5(7H)-one, **1a** is one of the intriguing indolopyridoquinazoline alkaloids isolated from ‘Wu-Chu-Yu’. Recent studies have demonstrated that rutaecarpine possess extensive biological and pharmacological properties, such as diuresis, perspiration, uterotonic action, improvement of cerebral functions, antinociception, inhibition of KCN-induced anoxia, specific 2,3,7,8-tetra- chlorodibenzo-*p*-dioxin binding, thermoregulatory and anti-obesity [4,5]. Among those, the cardiovascular pharmacological properties have attracted interest of the researchers. Rutaecarpine has been shown to have cardiovascular biological effects such as inotropic and chronotropic, vasorelaxant, anti-platelet aggregation and anti-inflammatory effects [6,7]. Furthermore, it has been reported that rutaecarpine has beneficial effects on some cardiovascular diseases [8,9,10]. This review summarized the cardiovascular pharmacological actions of rutaecarpine over the recent years, aiming to provide more evidence for rutaecarpine in the treatment of cardiovascular diseases.

## 2. The Cardiac Effects

Wu-Chu-Yu remains one of the useful drugs in the treatment of angina pectoris in China. As a major component of Wu-Chu-Yu, rutaecarpine (3 μM) has been reported to have transient positive inotropic and chronotropic effects on the guinea pig isolated right atria [6]. The above effect results from its activation of transient receptor potential vanilloid (TRPV1, also named capsaicin receptor), a receptor primarily expressed in sensory nerves innervating the heart and blood vessels. The activation of TRPV1 consequently induces the release of calcitonin gene-related peptide (CGRP), the principle transmitter in capsaicin sensitive sensory nerves.

Later studies reveal that CGRP also mediates the cardioprotective effects of rutaecarpine in some animal ischemia heart diseases models [8,9,10]. *In vitro,* rutaecarpine (1 or 3 μM) significantly enhances preservation with cardioplegia during reperfusion after hypothermic ischemia in isolated guinea pig hearts, characterized by improved recovery of cardiac function and reduced release of creatine kinase. Such effects are abolished by the competitive TRPV1 antagonist capsazepine, or by the selective CGRP receptor antagonist CGRP_8-37_ [8]. *In vitro,* in isolated guinea pig hearts, rutaecarpine (0.3 or 1 μM) exerts protective effects on cardiac anaphylaxis [9]. Rutaecarpine significantly improves cardiac function, decreases the content of tumor necrosis factor-alpha (TNF-alpha) in myocardial tissues concomitantly with markedly increasing the content of CGRP in the coronary effluent, which are depleted by CGRP (8-37) [9]. *In vivo*, pretreatment with rutaecarpine (100 or 300 μg/kg, i.v.) significantly reduces myocardial infarct size and creatine kinase release concomitantly with a significant increase in plasma concentrations of CGRP in myocardial ischemia-reperfusion rats [10]. Such effect is abolished by capsazepine or by pretreatment with capsaicin, which selectively depletes transmitters in capsaicin-sensitive sensory nerves [10].

Recently, it has been shown that capsaicin-sensitive sensory nerves contribute to the regulation of normal cardiac function and to the development of cardiac adaptation to ischemic stress [11]. Deletion of TRPV1 has been reported to exacerbate cardiac remodeling after myocardial infarction [12]. Our recent research found that rutaecarpine (10, 40 mg/kg. ig) reversed cardiac remodeling induced by isoprenaline. Isoprenaline significantly increased the ratio of left ventricle to body weight, the cross section area of cardiomyocyte, cardiac apoptosis and collagen deposition concomitantly with the decreased CGRP production, which were inhibited by treatment with rutaecarpine. The beneficial effects of rutaecarpine were abolished by pretreatment with capsaicin, which selectively depletes CGRP (data not published). These reports suggest that cardiac remodeling under pathological conditions is related to the dysfunction of sensory nerve and activation of sensory nerve may provide a novel therapeutic approach to reverse or delay the process of cardiac remodeling.

## 3. The Vasodilator/Depressor Effects

It has been demonstrated that rutaecarpine (0.1–10 μM) causes concentration-dependent relaxation of isolated rat superior mesenteric arterial and thoracic aorta segments precontracted with phenylepherine [13]. *In vivo*, the single dose injection of rutaecarpine (10–100 μg/kg i.v.) decreases the mean arterial pressure markedly [4]. 

Some reports reveal that rutaecarpine relaxes vascular smooth muscle through the activation of the endothelial Ca^2+^-NO-cGMP cascade and the inhibition of Ca^2+^ influx in vascular smooth muscle cells (VSMC), supported by the following evidence [4,14,15,16]: (1) The hypotensive effects of rutaecarpine (10–100 μg/kg, i.v.) are significantly inhibited by N^ω^-nitro-arginine (L-NNA), an NO synthase (NOS) inhibitor *in vivo* [15]. (2)The Ca^2+^-dependent vasorelaxant effects of rutaecarpine (0.1–3 μM) in rat thoracic aorta *in vitro* are significantly diminished by NOS inhibitors [N(G)-nitro-L-arginine methyl ester (L-NAME), L-NNA, L-N^G^-nitro-arginine], NO inactivator (hydroquinone), adenylate cyclase inhibitor (methylene blue) [14]. (3) In endothelial cells, rutaecarpine (0.1 and 1μM) augments [Ca^2+^]i, resulting in the release of NO and the potentiated cGMP production in aortic rings(1 μM). Denudation of endothelium significantly inhibits the effects of rutaecarpine [15]. (4) In VSMC, rutaecarpine (10 μM) inhibits voltage-dependent Ca^2+^ channels activity, resulting in decreased Ca^2+^ entry and [Ca^2+^]i and the decreased release of intracellular stored Ca^2+^ in VSMC. These two effects act simultaneously and result in vasodilatation [4].

It is recently suggested that the release and synthesis of CGRP via activation of TRPV1 mediates at least partly the depressor and vasodilator effects of rutaecarpine [17]. *In vitro*, rutaecarpine (0.1–10 μM) produces dose-dependent vasodilatation effect in isolated aortic and superior mesenteric rings with a concomitant increase of the CGRP concentration [17]. *In vivo*, intravenous injection of rutaecarpine (30, 100, or 300 μg/kg, i. v.) produces depressor effects with an increase in the plasma concentration of CGRP [17]. The above effects of rutaecarpine are significantly attenuated by capsaicin or CGRP_8-37_ [18].

Under some pathophysiological conditions, CGRP also mediates the antihypertensive effect of rutaecarpine [19,20,21,22]. *In vitro*, rutaecarpine (10 or 30 μM) inhibits vasoconstriction in presensitized guinea pig thoracic aorta challenged with antigen, which are significantly attenuated by CGRP_8-37_ [19]. *In vivo,* intravenous injection of rutaecarpine (30, 100, or 300 μg/kg, i. v.) causes dose-dependent hypotensive effects with a concomitant increase of plasma CGRP concentration in phenol-induced hypertensive rats, which is inhibited by capsaicin or capsazepine [20]. In the 2-kidney, 1-clip-induced hypertensive rats, continuous administration of rutaecarpine (10, 20, or 40 mg/kg/day, i.g) causes significant decrease in blood pressure with the increase in plasma CGRP concentration and the enhanced CGRP mRNA expression level in the dorsal root ganglion [21]. These effects of rutaecarpine are further confirmed in the spontaneously hypertensive rats [22].

It is suggested that the vasorelaxation induced by CGRP is endothelium dependent and NO stimulates CGRP release both *in vivo* and *in vitro* [23,24,25,26,27]. Therefore, it is reasonable to assume that rutaecarpine evokes CGRP release through activation of the NO pathway. However, the precise mechanism of the interactions between NO and CGRP in the pharmacological effects of rutaecarpine needs to be further investigated.

It is noteworthy that neither CGRP_8-37_ nor the inhibitor of NO block the vasodilator effects of rutaecarpine completely, which represents other unknown molecules may be also involved in the vasodilator effects of rutaecarpine. Prolylcarboxypeptidase (PRCP) is a serine protease that participates in the validated melanocortin metabolic pathway [28]. Very recently, it is reported that PRCP is another molecule that mediates the effects of rutaecarpine on the systolic blood pressure [29]. And rutaecarpine (10, 40 mg/kg. ig) inhibits mesenteric artery remodeling by increasing the protein or mRNA expression of PRCP in the circulation and small arteries [29].

## 4. Anti-Platelet Aggregation and Anti-Thrombus Activities

Intravascular thrombosis is one of the generators of a variety of cardiovascular disease and platelet aggregation is believed to play a crucial role in atherothrombotic processes. It is reported that indolopyridoquinazoline alkaloida including rutaecarpine have anti-platelet aggregation activity [30].

*In vitro*, rutaecarpine (40–200 μM) inhibits platelet aggregation in a dose-dependent manner stimulated by a variety of agonists [*i.e.*, collagen, ADP, adrenaline and arachidonic acid (AA)] in human platelet-rich plasma [31]. This effect of rutaecarpine is also verified in different animal models *in vivo*. Rutaecarpine (200 mg/g, i.v) inhibits platelet thrombi formation induced by irradiation of mesenteric venules with filtered light in mice pretreated intravenously with fluorescein sodium [5]. Rutaecarpine significantly prolongs the occlusion time, bleeding time, and the latent period of inducing platelet plug formation in mesenteric venules in rats, which is stronger than the positive control drug aspirin [5]. Furthermore, rutaecarpine is also effective in reducing the mortality of ADP-induced acute pulmonary thromboembolism in mice when administered intravenously [5].

Although human platelet releases NO and rutaecarpine exerts vasodilator effect via an NO dependent pathway, the antiplatelet aggregation activity of rutaecarpine is not significantly attenuated by pretreatment with the NOS inhibitor N(G)-monomethyl-L-arginine (L-NMMA) or L-NAME and with the guanylyl cyclase inhibitor methylene blue, which suggests that NO/cAMP pathway is not involved [31,32]. Interestingly, rutaecarpine significantly inhibits thromboxane B2 formation and inositol monophosphate formation stimulated by collagen and thrombin [31,32]. Further investigation reveals that those effects are due to the inhibition of phospholipase C activity, leading to reduced phosphoinositide breakdown, followed by the inhibition of thromboxane A2 formation, and then inhibition of [Ca^2+^]i mobilization of platelet aggregation stimulated by agonists [32].

Tissue factor (TF), also called platelet tissue factor, is necessary for the initiation of thrombin formation from the zymogen prothrombin [33]. Besides the above mechanism, the antiplatelet activity of rutaecarpine is related to the inhibition of the release of platelet-derived TF by stimulating the synthesis and releases of CGRP in SHR *in vivo* [22]. Taken together, all these results suggest that rutaecarpine has an effective anti-platelet effect both *in vivo* and *in vitro,* and be a potential therapeutic agent for arterial thrombosis.

## 5. Anti-Inflammation and Anti-Atherosclerosis

Inflammation is the basic response to a variety of external or internal insults. The release of inflammatory cytokines, such as prostacyclin (PG) E2 or PGD2, plays an important role in the inflammatory reaction [34]. Rutaecarpine (1–10 μM) reduces the production of PGE2 treated with lipopolysaccharide (LPS) or ultraviolet B (UVB) in a dose-dependent manner without changing the activity and expression of both cyclooxygenase (COX)-1 and COX-2 in cultured keratinocytes or RAW264.7 cells [7,35,36]. Furthermore, pretreatment of rutaecarpine also significantly decreases calcium ionophore A23187 induced-PG production and [3H]-AA release [35]. Steroidogenesis is another mechanism responsible for the inflammation. It is recently reported that rutaecarpine (100 μM) inhibits corticosterone production in the rat zona fasciculata-reticularis (ZFR) cells via decreasing the activity of cAMP and steroidogenic enzymes [37]. *In vivo*, application of biomimetic mixture of synthetic and natural forms of the active components of Evodia fruit extract, containing rutaecarpine, dehydroevodiamine, and evodin (0.1-1%) significantly inhibits the localized erythema induced by methyl nicotinate [38]. All these suggest that anti-inflammatory effect of rutaecarpine is, at least in part, ascribed to the diminution of PGs production through inhibition of AA release.

It has been known that reactive oxygen species (ROS) and the resultant damage to lipids, proteins and DNA and the changed gene expression play an important role in the pathogenesis of inflammation. NADPH oxidase is the major ROS-producing enzyme. It has been shown that rutaecarpine inhibits bacterial endotoxin (e.g., LPS) or cytokines (e.g., interferon-γ)-stimulated production of NO and ROS in microglial cells, one of the most important type of effectors cells in mediating central nerve system (CNS) inflammatory responses [39]. Rutaecarpa and its four bioactive components all exhibit anti-inflammatory activities which could be partially explained by their different potentials for inhibiting NADPH oxidase-dependent ROS and/or iNOS-dependent NO production in activated inflammatory cells [39].

The changed gene expression induced by excessive production of ROS is crucial in the inflammation. Matrix metalloproteinase (MMP) is a key component in photoaging of the skin due to exposure to ultraviolet A (UVA). In cultured human keratinocytes, irradiation increases both the gelatinolytic activities and the protein and mRNA expression of MMP-2 and MMP-9, which is due to the excessive production of ROS. Rutaecarpine decreases the UVA -induced increased generation of ROS and the increased expression of MMP-2 and MMP-9 [40]. These results further suggest that rutaecarpine may be useful in the prevention of ultraviolet A-induced photoaging.

Atherosclerosis, the most common cardiovascular disease, is now defined as a chronic inflammatory disease. LIGHT (a receptor expressed by T lymphocytes/tumor necrosis factor-related 2) belongs to a member of the TNF ligand superfamily and acts as a new player in the atherogenesis [41]. It is reported that rutaecarpine (10 μM) has inhibitory effects on LIGHT-induced migration and the activation of CCR1, CCR2, ICAM-1, ERK, and p38 MAPK in the monocyte-like cell line THP-1 and promyelocytic leukemia cell line HL60 [42]. This effect is also involved in the decreased ROS production and NADPH oxidase activation. These results indicate that rutaecarpine has the potential for use as an anti-atherosclerosis agent.

## 6. Anti-Obesity and Potential Anti-Diabetic Metabolic Syndrome

Rutaecarpine (20 or 100 mg/kg, i.p.) was recently reported to ameliorate bodyweight gain, which is related to the inhibition of orexigenic neuropeptide Y and agouti-related protein [43]. These two neuropeptides play major roles in feeding and are closely related to obesity and diabetic metabolic syndrome. The above results suggest that rutaecarpine has potential effects on diabetic metabolic syndrome.

## 7. Conclusions

It has been suggested that, for the future drug development, herbs may be an important source of new compounds which may have fewer side effects. Rutaecarpine is a pure chemical isolated from Wu-Chu-Yu. The understanding of the precise pharmacological properties of rutaecarpine will definitely help the development of this new drug. The review summarizes the studies on the cardiovascular effects of rutaecarpine, which provides extensive and strong evidence for the potential use of this promising drug in the treatment of cardiovascular diseases.

Up to now, modifications of rutaecarpine structure have been investigated for a long time and the potential structure-activity relationship has been studying. Some sites have been indicated to be the key sites for certain activities. Recently, it is reported that the 14-N atom of rutaecarpine might be the key site for the vasodilator and hypotensive activity, which is very different from the traditional agonists of TRPV1 [44]. These results suggest that this novel class of enhancers could be a new type of pharmacological activity for activating TRPV1 and rutaecarpine can be a potential anti-hypertensive leading compound with novel mechanism. 

It has been shown that the dissolubility of rutaecarpine is very low, which affects its absorption efficiency. It has been suggested that the solid dispersion of rutaecarpine produces anti-hypertensive effect in SHR, while crude rutaecarpine has very poor effects at the same doses [45]. This kind of attempt will increase the druggability of rutaecarpine. 
